# A cross-sectional study exploring general practitioners’ views on dietary supplements

**DOI:** 10.1186/s12875-024-02654-4

**Published:** 2024-11-26

**Authors:** Sophia Wagner, Sascha Eickmann, Hansjörg Baurecht, Anne Herrmann

**Affiliations:** 1 Department of Epidemiology and Preventive Medicine, Medical Sociology, University Regensburg, Regensburg, Germany; 2 Department of Epidemiology and Preventive Medicine, University Regensburg, Regensburg, Germany; 3 School of Medicine and Public Health, University of Newcastle, Newcastle, Australia

**Keywords:** Dietary supplements, General practice, Cross-sectional, Survey, CAM

## Abstract

**Background:**

Dietary supplements (DS) are often used by patients to enhance their health and well-being. General practitioners (GPs) are commonly the first point of contact for patients who enquire about DS. The aim of this study was to explore GPs’ views on DS.

**Methods:**

A cross-sectional purposeful recruitment online survey of 162 general practitioners (GPs) in Germany was conducted between May and August 2021. The questionnaire assessed GPs’ views on dietary supplements (DS), including perceptions of safety, efficacy, and importance in medical practice. Data were analyzed using inferential statistics and logistic regression analyses to explore associations between GPs’ views and demographic factors.

**Results:**

Response rate could not be determined because multipliers were included here by means of personal networks, consent rate was 100%. Many respondents considered DS to be an important topic in their daily practice (64,8%, *n* = 99). Almost two thirds were convinced of their efficacy and considered DS to be safe for use (61,2%, *n* = 93). However, the majority of respondents were in favor of more standardized guidelines (86.8%, *n* = 132) and improved medical education on the handling of DS in routine care (89,5%, *n* = 136). Physicians who self-administered DS were statistically significantly more likely to perceive them as safe to use, with an OR of 4.25 (95% CI: 1.74–10.40). Self-administration [OR 4.52 (1.67–12.22)] and participation in continuous medical education (CME) [OR 3.52 (1.133–9.38)] were positively associated with perceiving them as an important topic.

**Conclusions:**

To the best of our knowledge, this is the first study to assess German GPs’ perceptions regarding DS. The majority of physicians recognized the importance of DS in routine care but wished for improved regulation and more standardized guidelines regarding their use. The findings could be used to develop targeted educational interventions and improve handling of DS in daily general practice.

**Supplementary Information:**

The online version contains supplementary material available at 10.1186/s12875-024-02654-4.

## Key messages

General practitioners (GP) in our study see dietary supplements (DS) as an important matter in their daily practice. While many GPs perceive DS to be safe for their patients, most of them wish for stricter regulations and guidelines on handling their use by patients. Practically all GPs expressed a need for enhanced education on DS.

**Supplementary Information:**

The online version contains supplementary material available at 10.1186/s12875-024-02654-4.

## Introduction

The global dietary supplements (DS) market has been growing steadily in the past decades and Germany is no exception to this trend. With sales reaching nearly 3 billion euros in 2022 [1], a substantial number of adults are regularly turning to vitamins and minerals as DS to support their health and wellbeing [2]. With the growing interest in DS, there has been an increasing amount of research assessing their potential effects and side-effects [3]. While some studies have shown positive effects on patient outcomes, others suggest that the use of DS should not be taken lightly or without guidance by physicians [4]. A wide range of side-effects and hepatotoxic properties have been associated with certain DS [3], such as high doses of vitamins E and A, which may contribute to the promotion of bronchial carcinomas [5]. Despite potential risks DS are currently classified as “food” in Germany and are therefore subject to less stringent regulatory measures [4, 6]. They are available over-the-counter (OTC) and are generally perceived as safe by patients and clinicians [4, 7]. The increased public attention and popularity that DS are seeing over the last couple of years, e.g. on social media, are currently not reflected, i.e. mentioned in relevant practice guidelines, including the GP guideline polypharmacy of the German Society for General Practice and Family Medicine [8] and guidelines for check-up appointments [9]. GPs are often the first point of contact for patients, who confront them with inquiries and uncertainties about DS [10]. Given the increasing popularity of DS, providing patients with adequate information and support, enabling them to better understand the potential benefits and risks of DS. This makes it all the more important to overcome hopes and misinformation, some of which are difficult to fulfill [11]. Nevertheless, there is a lack of knowledge on GPs’ views of patients’ consumption of DS [12]. While various international studies have examined healthcare professionals’ attitudes towards and communication practices concerning DS [12–14], their findings may not be applicable to the German healthcare system due to variations in regulatory and clinical practices [6, 15]. To address this gap, our study aims to investigate the perceptions of German GPs on DS with the aim to develop strategies for how to improve handling of DS in general practice and provide optimal patient-centered healthcare.

## Methods

### Design

This was an explorative cross-sectional survey study using an online questionnaire, developed by an interdisciplinary team of healthcare service researchers, physicians, and nutritionists, based on literature review [12, 13]. The questionnaire underwent a two-phase pre-testing with six physicians. The Flesch-Kincaid test was used to assess readability with a level of 10 considered to be appropriate. In the pretest phase adjustments were made in terms of comprehensibility, wording, clarity, logic and spelling in order to increase internal validity. We assessed the internal consistency of the questionnaire using Cronbach’s alpha reliability coefficient. Items related to ‘Attitudes towards DS’ (‘DS are safe to use’, ‘DS do more harm than benefit’, ‘DS can be a good alternative to conventional prevention and therapy methods’, ‘DS only help through placebo effects’, ‘DS are an important medical topic’) and ‘Attitudes towards Regulation’ (‘DS must be regulated more strictly’, ‘There should be more uniform regulation in the handling of DS in everyday medical practice’, ‘More attention should be paid to DS in the medical curriculum’) were grouped accordingly. The Cronbach’s alpha for the ‘Attitudes towards DS’ scale was 0.772, indicating acceptable internal consistency. The ‘Attitudes towards Regulation’ scale had a Cronbach’s alpha of 0.702, suggesting acceptable reliability (see Table 1). In addition, data was collected on the socio-demographics of the GPs as well as their training and practice experience, including additional qualifications, practice types and location.

**Table 1:** Internal consistency

Group	Items	Cronbach’s alpha
*Attitudes towards DS*	DS are safe to use	0.772
	DS do more harm than benefit	
	DS can be a good alternative to conventional prevention and therapy methods	
	DS only help through placebo effects	
	DS are an important medical topic	
*Attitudes towards regulation*	DS must be regulated more strictly	0.702
	There should be more uniform regulation in the handling of DS in everyday medical practice	
	More attention should be paid to DS in the medical curriculum	

### Eligibility criteria

To be eligible for this study, participants had to self-report as working GPs in Germany. Residents self-reporting work in both GP practices and hospitals were also included in the study.

### Recruitment and data collection

Participants were purposefully recruited between May and August 2021 via lists of general medical teaching practices at various German universities and personal networks. Due to the recruitment within personal networks and other stakeholders associated with GPs a response rate cannot be calculated. An online questionnaire was used to recruit respondents for a cross-sectional analysis. Digital consent was obtained in advance in accordance with the European general data protection regulations [16]. The principles of the Declaration of Helsinki were adhered to and the local ethics committee provided approval for this body of work (authorization No: 21-2310-101). The survey data were pseudonymized. To ensure GDPR compliance the email addresses of participants who wished to receive study results were collected in a separate database from the main questionnaire data.

### Data evaluation and statistical analysis

The study sample was analyzed descriptively. We conducted inferential statistical analyses including chi-squared tests to assess associations between categorical variables, acknowledging that relationships between perceptions and other variables are inherently bivariate in nature. Logistic regression models were used to assess associations between variables. Dependent variables such as physician’s subjective assessment about DS safety, DS only work as a placebo and DS as an important medical topic was measured on a 4-point likert scale (agree, rather agree, rather disagree, disagree). Independent variables were age, sex, specialist, naturopath qualification (yes/no), practical experience (< 10, 11–20, 21–30, 31–40, > 40 years), location of practice (rural and small town or big town), practice form (solo or shared practice), physician’s self-administration of DS (yes/no) and CME for DS (yes/no). All variables with more than 2 categories were dichotomized into agreement vs. disagreement. Goodness-of-fit for logistic regression models was evaluated using the Hosmer-Lemeshow test, with a p-value greater than 0.05 indicating adequate fit. As sensitivity analysis we evaluated the impact of age and sex by testing for interactions. In addition, we examined subgroups stratified by duration of high experience level (≤ 10 versus > 10 years) and binary self-administration of DS. A comprehensive evaluation of the model was carried out, which included goodness-of-fit test. All statistical tests were two-sided and P-values < 5% were considered significant. Analyses were conducted using IBM SPSS Statistics (version 26.0.0.0).

## Results

### Sample characteristics

162 participants completed the survey, this equated to a 100% consent rate. On average they were 50 years old and 55% of them were male. 83.5% were GPs, the remaining respondents were from other specialties that are also eligible to practice as GPs in Germany, such as specialty degree in internal medicine.

About 40% of participants reported to consume DS themselves. Further information on practice location, professional experience and practice form are presented in Table 2.

**Table 2:** Sample characteristics

Characteristics *n* = 162		
	**n**	$\emptyset$ **(SD)**
*Age (Mean)*	137	50.24 (11.13)
NA	25	
	**n**	**in %**
*Gender*	147	
female	64	43.5
male	81	55.1
various NA	2 15	1.4
*Specialist*	146	
yes	124	84.9
no NA	22 18	15.1
*Medical specialist designation*	127	
General practitioner	106	83.5
Medical Specialist for internal medicine GP and internal medicine	17 3	13.4 2.4
Other specialist NA	1 35	0.8
*Additional title specialization in alternative medicine /naturopathic treatment*	162	
yes no NA	25 137 0	15.4 84.6
*Practical Experience* *(in years)*	140	
up to 10	31	22.1
11–20	31	22.1
21–30	41	29.3
31–40	32	22.9
above 40 NA	5 22	3.6
*Federal state* Baden-Wuerttemberg Bavaria Bremen Lower Saxony NA	146 38 104 1 3 16	26.0 71.2 0.7 2.1
*Practice location* *(according to inhabitants)*	138	
Large city (> 100.000)	21	15.2
Small town (> 20.000)	34	24.6
Country (< 20.000)	83	60.1
NA	24	
*Form of practice*	146	
Individual practice	48	32.9
Joint practice	76	52.1
Ambulatory health care center	9	6.2
Hospital NA	13 16	8.9
*Self-reported use of dietary supplements*	151	
yes	62	41.1
no NA	89 11	58.9

### Views of GPs on DS

61% of respondents (*n* = 93) reported that DS are safe to use. 26.2% (*n* = 40) believed DS only produce placebo effects. Nearly two-thirds of participants considered DS an important medical topic (*n* = 99). However, 78.2% (*n* = 118) felt DS should be more strictly regulated. 86.8% of respondents (*n* = 132) supported the implementation of more standardized guidelines for DS in medical practice. 90% (*n* = 126) of respondents expressed a desire for improved medical education on DS. The highest recommendation for DS was seen with vitamins (60%), closely followed of minerals (58%). The data is presented in Table 3. Half of GPs reported regularly participating in continuous medical education (CME) on DS (50.0%, *n* = 76). Half of these (50%) expressed dissatisfaction with the current quantity of courses related to this topic. Additionally, 53.4% were not convinced by the presented evidence or implications for practice. 45,8% of GPs reported to discuss DS with colleagues on a regular basis (Table 4).

**Table 3:** Attitudes of GPs towards DS

	*n*	in %
*DS are safe to use.*		
**Mean (SD)** Total 1 agree 2 rather agree 3 rather disagree 4 disagree NA	**2**,**37** 152 17 76 44 15 10	**(0**,**812)** 100 11.2 50.0 28.9 9.9
*DS only help through placebo effects.*		
**Mean (SD)** Total 1 agree 2 rather agree 3 rather disagree 4 disagree NA	**3**,**05** 153 5 35 61 52 9	**(0**,**838)** 100 3.3 22.9 39.9 34.0
*DS are an important medical topic.*		
**Mean (SD)** Total 1 agree 2 rather agree 3 rather disagree 4 disagree NA	**2**,**27** 153 29 70 38 16 9	**(0**,**889)** 100 19.0 45.8 24.8 10.5
*DS need to be regulated more.*		
**Mean (SD)** Total 1 agree 2 rather agree 3 rather disagree 4 disagree NA	**1**,**95** 151 46 72 28 5 11	**(0**,**790)** 100 30.5 47.7 18.5 3.3
*There should be more uniform guidelines on the handling of DS in everyday medical practice.*		
**Mean (SD)** Total 1 agree 2 rather agree 3 rather disagree 4 disagree NA	**1**,**76** 152 61 71 15 5 10	**(0**,**761)** 100 40.1 46.7 9.9 3.3
*More attention should be paid to DS in the study.*		
**Mean (SD)** Total 1 agree 2 rather agree 3 rather disagree 4 disagree NA	**1**,**67** 152 69 67 13 3 10	**(0**,**717)** 100 45.4 44.1 8.6 2.0

**Table 4:** CME modalities of GPs towards DS

Attending DS training courses yes no NA	152 76 76 10	50.0 50.0
*Sufficient amount and range of courses* yes no NA	74 37 37 2	50.0 50.0
*Satisfaction with content of training courses* yes no NA	73 34 39 3	46.6 53.4
*Exchange with colleagues* yes no NA	153 70 83 9	45.8 54.2

### Association of views with socio-demographic, training and practice-related factors

Results of the logistic regression analyses indicated that several factors influence physicians’ views on DS, such as personal use of DS (Table 4). Physicians who reported to self-administer DS were significantly more likely to perceive them as safe to use with an odds ratio (OR) of 4.25 (95% CI: 1.74–10.40). They were also more likely to consider DS an important medical topic, as indicated by an OR of 4.52 (95% CI: 1.67–12.22). Specialists were found to be less likely to view DS as mere placebo effects [OR 0.17 (0.04–0.84)] compared to non-specialists. In our sample, GPs who favored stricter regulation of DS were more likely to be practicing in solo practice [OR 0.22 (0.06–0.82)]. GPs practicing in rural areas were less likely to favor uniform regulations compared to those practicing in urban areas [OR 0.09 (0.02–0.45)].

Finally, specialists were found to be more inclined towards incorporating DS education in medical training [OR 7.66 (1.17–50.06)]. Participation in CME on DS [OR 3.52 (1.33–9.38)] was positively associated with perceiving DS as an important medical topic. Sex was significant for the perception that DS are safe to use (Table 4). Furthermore, effects between age and specialist status showed no significant associations. The data for this section is shown in Table 5.

**Table 5:** Logistic regression models between attitudes and socio-demographic, practice-related factors and training. Reported in OR, in brackets the 95% confidence interval, * *p* < 0.05

	DS are safe to use.	DS only help through placebo effects.	DS are an important medical topic.	DS must be regulated more strictly.	There should be more uniform regulation in the handling of DS in everyday medical practice.	More attention should be paid to DS in the medical curriculum.
Age	1.05 (0.961–1.146)	0.96 (0.86–1.08)	1.03 (0.94–1.13)	1.02 (0.93–1.11)	0.99 (0.89–1.09)	0.93 (0.83–1.05)
Sex	0.49* (0.16-0.99)	0.53 (0.19–1.45)	0.93 (0.36–2.43)	1.40 (0.50–3.93)	2.02 (0.59–6.95)	3.82 (0.76–19.16)
Specialist	3.31 (0.75–14.57)	0.171* (0.04–0.84)	1.74 (0.35–8.74)	1.20 (0.18–8.23)	5.43 (0.91–32.45)	7.66* (1.17–50.06)
Naturopath qualification	1.06 (0.33–3.34)	0.28 (0.06–1.42)	1.44 (0.43–4.79)	3.55 (0.78–16.18)	5.44 (0.56–52.93)	1.10 (0.11–10.63)
Experience	0.92 (0.43–1.99)	1.23 (0.51–3.23)	1.57 (0.72–3.42)	0.98 (0.440–2.18)	1.28 (0.51–3.23)	3.07 (0.99–9.51)
Location of practice	0.93 (0.46–1.88)	1.24 (0.56–2.75)	1.08 (0.52–2.26)	0.93 (0.65–1.33)	0.09* (0.02–0.45)	1.12 (0.79–1.59)
Practice form	1.18 (0.59–2.36)	1.6 (0.61–4.60)	1.32 (0.64–2.75)	0.22* (0.06–0.82)	1.09 (0.76–1.56)	0.87 (0.61–1.25)
Self-administration	4.25* (1.74–10.40)	0.33* (0.12–0.91)	4.52* (1.67–12.22)	0.42 (0.15–1.17)	0.42 (0.13–1.36)	1.56 (0.10–23.50)
CME for DS	1.07 (0.46–2.49)	0.69 (0.27–1.79)	3.52* (1.33–9.38)	0.57 (0.20–1.58)	0.77 (0.23–2.60)	5.21 (0.81–33.55)

## Discussion

Many of our respondents believed in the effectiveness of DS and considered them safe to use. However, the association between sex and the belief that DS are safe to use suggests that female GPs may have different perspectives on DS safety compared to male GPs. This difference could be due to varying personal experiences, patient interactions, or levels of engagement with DS. Our analyses suggest that the variables’ influence on GPs’ views of DS operates independently rather than synergistically, as demonstrated particularly by the distinct OR for sex regarding DS safety. Further research is needed to explore these differences. Overall, the majority of physicians acknowledged the significance of DS but desired improved regulation and more uniform policies regarding their use. Our findings also highlight the significant role of personal use of DS in shaping physicians’ views. Demographic factors and professional experience also seem to impact on GPs’ views, with GPs practicing in rural areas favoring a less strict regulation of DS compared to those practicing in urban areas. Our findings highlight the need to better integrate DS into medical education.

### Call for more rigorous regulation of dietary supplements

60% of our study participants believed that DS are safe to use. However, the majority of our respondents would like to see more regulation in the approval processes for DS. Livingston et al. (2010) reported that only 25% of GPs in Australia considered DS to be safe to use. Australian GPs were most concerned about side-effects, quality, and lack of evidence on DS [12]. Stronger regulation, for example closer to the approval criteria for regular medication, could reduce physicians’ uncertainties related to DS [17]. Stronger regulations have also been called for in a recent review [4, 18].

In the US DS are regulated according to the ‘Current Good Manufacturing Practices (cGMPs)’, which are monitored by the FDA. The FDA has the authority to penalize manufacturers or products that do not comply with these standards [19]. In the EU however, DS are regulated differently from newly authorized or established medicines. DS fall under the ‘Food Supplements Directive (2002/46/EC)’, but they are not controlled by the European Medical Agency (EMA) [20]. Therefore, individual producers cannot be subject to any bans or conditions.

Our data indicate that most GPs would like to see more uniform guidelines for the use of DS in everyday medical practice. Especially experienced physicians (specialists) recommended guidelines for the use of DS to increase patient safety and satisfaction, which is associated with more GPs’ confidence to deal with this topic [17]. A step in this direction has already been taken by the German General Practitioner’s Association (DEGAM). They reported that an initial guideline on the topic of “Advice on vitamin D substitution” is in progress and should be completed by the end of 2024 [8].

### Increasing awareness and relevance of dietary supplements for medical practice

There is evidence to suggest a rise in interest towards self-improvement of health and wellbeing, specifically in enhancing the immune system and optimizing the body’s response to everyday stress, at work or in sports [21]. Notably, patients interest in DS has been stimulated by COVID-19 “to boost the immune system” and was further stimulated by influencer across various social networks, targeting especially younger people [22]. Previous research has suggested that if physicians do not take the issue of supplement use seriously or do not consider it important to address with patients, the likelihood of patients concealing their supplement use decreases significantly [23, 24].

Also, many GPs in our study reported to take DS themselves. Our data could show that there is a higher chance of physicians taking DS themselves, consider DS an important topic (OR 4.5) and safe to use (OR 4.25). Nearly two-thirds of them considered DS to be medically relevant. Only 26.2% of them believed that DS works through placebo effect. This perception is countered by evidence that DS might be harmful [4]. Our results confirm previous studies that demonstrate a conflicted attitude toward DS, oscillating between favor and skepticism [15]. This could mean that there is an evidence-practice gap, which hasn’t been fully addressed yet [25].

### Desire for more teaching and training

In our survey, the majority of respondents indicated that they would like to see more medical education on DS, which also leads to an increased awareness of how important this topic might be for the daily routine. For some time now, the Federal Association of Medical Students in Germany (BVMD) has been calling for more teaching on nutritional aspects in medical studies, which shows that this topic area may still be inadequately addressed by current curricula [26]. Our data could show that specialists find this topic more important. It is associated with a OR of 7.6 that specialists wish for a stronger foundation in the medical curriculum. The Association for Nutrition of England integrated a newly developed curriculum for nutritional medicine in medical school [27]. Only about half of the participants in our study indicated that they attend CME on this topic. Only about 50% of them were satisfied with the number and the content of the courses. Other studies are consistent with our findings, as it has been stated that there are few satisfactory courses available and that more CME on DS is desired [28]. More efforts are required to improve education and training in DS, e.g. by providing better structured content or engaging with small-group, problem-based learning [29].

### Future research directions

Exploring the reasons for the views of GPs on DS, e.g. why GPs take DS themselves or why there is a visible gradient between policy demands on rural and urban practices might explain some of our findings further and might be useful to tailor future CME. In addition, future research should investigate the knowledge and communication practices of GPs in relation to DS to help develop strategies to improve care on this area.

Based on our findings, we consider it desirable to pay more attention to DS in medical studies, and to review the training courses on DS and, if necessary, improve and expand the content and number of courses.

## Conclusion

This is the first pilot cross-sectional study to assess the views of German GPs on DS. A significant number of surveyed physicians trust in the efficacy and safety of DS. The majority emphasizes the importance of DS. However, they call for stricter regulation and more consistent guidelines for their use. Our study also highlights how personal use of DS shapes doctors’ attitudes. Demographic characteristics and professional experience can influence the views of GPs. Those in rural areas generally favor more lenient regulation of dietary supplements compared to their urban counterparts. These findings highlight the importance of integrating dietary supplements more thoroughly into medical education. This could encourage GPs to adequately introduce and use dietary supplements with their patients and provide them with accurate information about their safety and efficacy. Further studies are necessary to provide a better and more systematic picture.

### Strength and limitations

There is a lack of data on the views of GPs in Germany regarding DS.

The study’s data was collected from GPs in only some German federal states, limiting the generalizability of the findings. Data collection took place during the COVID-19 pandemic in spring/summer 2021, when DS may have played a greater role due to the unavailability of vaccines and the common use of DS for immune support [30]. Selection bias may have occurred, as physicians who use DS themselves or have a greater interest in DS/nutrition might have been more likely to complete the survey. The recruitment process was not systematic, further contributing to potential selection bias. This study highlights preferences and beliefs about DS among GPs and identifies areas for future research that require more thorough investigation.

## Electronic supplementary material

Below is the link to the electronic supplementary material.

Supplementary Material 1

## Data Availability

The data can be viewed on reasonable request.
