# Supplementary material for: A cross-sectional study exploring general practitioners’ views on dietary supplements
Source: BMC Prim Care. 2024 Nov 26;25:401. doi: 10.1186/s12875-024-02654-4 (PMC11590300; doi:10.1186/s12875-024-02654-4)
Supplement: Supplementary file 1 — Supplementary Material 1 [file 12875_2024_2654_MOESM1_ESM.docx]

NUTRITIONAL SUPPLEMENTS IN

GENERAL MEDICINE

Dear colleagues,

thank you very much for your interest in our study and for taking part in our survey!

Our study focusses on the topic of dietary supplements (NEM) in general practice.

The following questionnaire is divided into three parts:

Knowledge about food supplements, attitudes towards food supplements and

communication about food supplements during general medical check-ups.

With this survey, we want to gain insights into the relevance and handling of

nutritional supplements in everyday general practice and obtain results that will benefit you and your patients.

The survey will take approx. 10 - 12 minutes to complete.

All information you provide will be used exclusively for scientific purposes, will be treated confidentially and will not be passed on to third parties. After data collection, all data will be pseudonymised so that the data cannot be traced back to you personally. All data collected from the survey will be summarised in a database for analysis. The results of the survey will be published anonymously.

The study is being conducted under the direction of Sascha Eickmann (MSc.) and Prof. Dr Anne Herrmann-Johns at the Institute of Epidemiology and Preventive Medicine, Medical Sociology at the University of Regensburg .

Thank you very much in advance for your time and effort!

Sophia Wagner

University of Regensburg

Institute of Epidemiology and Preventive Medicine

Medical Sociology

Dr.-Gessler-Straße 17

93051 Regensburg

Sascha Eickmann

Phone: XXXX

E-Mail: XXXX

I have read the information about the data processing procedure and agree to take part in the following survey. I agree to the storage of my data for the specified period.

| □ **yes** | □ **no** |
| --- | --- |

**KNOWLEDGE**

Dear participants, the first part of the survey is about categorising your knowledge of dietary supplements.

**How would you rate your own knowledge (in school grades) of food supplements?**

| **1** | **2** | **3** | **4** | **5** | **6** |
| --- | --- | --- | --- | --- | --- |
| □ | □ | □ | □ | □ | □ |

**Which food supplements (or derivatives) do you recommend without hesitation?**

*(Multiple selection possible)*

| □ Vitamins (e.g. Vitamin D) |  |  |
| --- | --- | --- |
| □ Minerals (e.g. Magnesium) |  |  |
| □ Fats (e.g. Omega-3-FS) |  |  |
| □ Other: |  |  |

**How confident do you feel about working with food supplements?**

| **very confident** | **confident** | **rather confident** | **rather uncertain** | **uncertain** | **very uncertain** |
| --- | --- | --- | --- | --- | --- |
| □ | □ | □ | □ | □ | □ |

**Do you have any reliable sources of information on food supplements?**

| □ **yes** | □ **no** |
| --- | --- |

- *If you picked yes, which ones? (Multiple selection possible)*

| □ Books | □ Journals | □ Databases  (e.g. Pubmed) | □ Clinical decision systems  (e.g. Uptodate) |
| --- | --- | --- | --- |

| □ Webpages | | □ Pharmaceutical decision systems | □ opinions of colleagues |
| --- | --- | --- | --- |
| □ Other  _______________  _______________ |  |  |  |

In the following section you will find questions on the subject of food supplements to help you categorise your knowledge of food supplements. We would ask you not to use any aids. If you cannot answer a question, you can also skip it.

1. **Before being launched on the market, are food supplements reviewed in the same way as drugs?**

| □ | True |
| --- | --- |
| □ | False |

1. **Which vitamin supplements are used most frequently on average in Germany?**

| □ | Vitamin C and E |
| --- | --- |
| □ | Vitamin D and A |
| □ | Vitamin B12 and C |
| □ | Vitamin B6 and A |

1. **How many people in Germany regularly take food supplements on average?**

| □ | 5-10% |
| --- | --- |
| □ | 10-25% |
| □ | 20-50% |
| □ | 40-60% |

1. **Which patient groups have been shown to take food supplements more frequently?** (select **one option** per line)

| **Age:** | □ | Teenagers | □ | Adults |
| --- | --- | --- | --- | --- |
| **Sex:** | □ | Women | □ | Men |
| **Physical activity:** | □ | Physically active people | □ | Physically inactive people |
| **Education level:** | □ | People with a high level of education | □ | People with a low level of education |

1. **Do many people in Germany have a clinically relevant folic acid deficiency?**

| □ | Yes |
| --- | --- |
| □ | No |

1. **Which vitamins, minerals or their derivatives have been shown to promote lung cancer in case of overdose?**

| □ | Vitamin D and B2 |
| --- | --- |
| □ | Vitamin A and E |
| □ | Vitamin C and B6 |
| □ | Vitamin E and C |

1. **What symptoms can a potassium overdose trigger?**

| □ | Cardiac arrhythmia, kidney stones, vomiting |
| --- | --- |
| □ | Cardiac arrhythmia, paraesthesia, muscle weakness |
| □ | Seizures, pancreatitis, diarrhoea |
| □ | Seizures, vomiting, psychotic states |

1. **Which symptoms can be caused by Vitamin B6 deficiency?**

| □ | Night blindness |
| --- | --- |
| □ | Pagophagia (ice addiction) |
| □ | Acrodermatitis |
| □ | Tetany |

1. **What morbidities has vitamin C supplementation been proven to have a positive effect on?**

| □ | Cancer |
| --- | --- |
| □ | CHD |
| □ | Dementia |
| □ | None of the diseases mentioned |

1. **How are food supplements defined?**

| □ | Products intended to supplement the normal diet |
| --- | --- |
| □ | Drugs intended to supplement the normal diet |
| □ | Products intended to compensate for low vitamin and mineral levels |
| □ | Drugs intended to compensate for low vitamin and mineral levels |

**ATTITUDE**

The following part consists of questions about your attitude towards food supplements.

**Do you take dietary supplements yourself?**

| □ **yes** | □ **no** |
| --- | --- |

- If **yes**: How often?

| **Daily** | **Several times a week** | **Weekly** | **Several times a month** | **Once a month or less often** |
| --- | --- | --- | --- | --- |
| □ | □ | □ | □ | □ |

- Which dietary supplements do you take?

| □ Vitamins: | e.g. Vitamin D |  |  |
| --- | --- | --- | --- |
| □ Minerals: | e.g. Magnesium |  |  |
| □ Fats: | z.e.g Omega-3-fatty acids |  |  |
| □ Other: |  |  |  |

**Do you discuss food supplements with colleagues?**

| □ **yes** | □ **no** |
| --- | --- |

- If **yes:**

How do you exchange ideas with colleagues?

| □ informal discussion  □ CME | □ Conferences  □ Online platforms | □ Other:  ________________________ |
| --- | --- | --- |

**Do you participate in further training opportunities (e.g. via journals) on food supplements?**

| □ **yes** | □ **no** |
| --- | --- |

- If **yes:**

**How many organised training courses on this topic have you attended in the last year?** (also online)

**Are there enough training courses on the subject of food supplements?**

| □ **yes** | □ **no** |
| --- | --- |

**Are you satisfied with the training programmes?**

| □ **yes** | □ **no** |
| --- | --- |

**Where do you see room for improvement for further training in the field of nutritional supplements?**

**How do you view the following statements?**

|  |  | **Fully agree** | **Agree somewhat** | **Rather disagree** | **Do not agree** |
| --- | --- | --- | --- | --- | --- |
| 1. | Food supplements are safe to use. | □ | □ | □ | □ |
| 2. | Food supplements do more harm than  benefit. | □ | □ | □ | □ |
| 3. | Food supplements can be a good alternative to conventional prevention and therapy methods. | □ | □ | □ | □ |
| 4. | Food supplements only help through the  placebo effect. | □ | □ | □ | □ |
| 5. | Food supplements are an important  medical topic. | □ | □ | □ | □ |
| 6. | Food supplements must be  regulated. | □ | □ | □ | □ |
| 7. | There should be more standardised regulation for dealing with food supplements in everyday medical practice. | □ | □ | □ | □ |
| 8. | More attention should be paid to food supplements in medical school. | □ | □ | □ | □ |

**HEALTH AND CANCER SCREENING / CHECK-UPS**

The last part of the survey deals with communication between you and your patients on the subject of food supplements.

**How many check-ups (health and cancer screening) do you carry out yourself each year?**

__________________________________________________

(approximate number in absolute figures e.g. 100)

**On average, how much time can you devote to a patient's preventive healthcare?** (in minutes)

| **<10** | **10** | **15** | **20** | **> 25** |
| --- | --- | --- | --- | --- |
| □ | □ | □ | □ | □ |

- **How much time do you spend on the anamnesis or a consultation?** (in %)

| **100 – 90 %** | **90 – 75 %** | **75 – 50 %** | **50 – 25 %** | **25 – 10 %** | **10 – 0 %** |
| --- | --- | --- | --- | --- | --- |
| □ | □ | □ | □ | □ | □ |

**On average, how often do you talk about … during check-up appointments?** (in %)

|  | **100 – 90 %** | **90 – 75 %** | **75 – 50 %** | **50 – 25 %** | **25 – 10 %** | **10 – 0 %** |
| --- | --- | --- | --- | --- | --- | --- |
| … Nutrition | □ | □ | □ | □ | □ | □ |
| … Traditional medicine | □ | □ | □ | □ | □ | □ |
| … Dietary supplements | □ | □ | □ | □ | □ | □ |
| … Exercise/ sport | □ | □ | □ | □ | □ | □ |

**How often do you actively ask for … during the interview?** (in %)

|  | **100 – 90 %** | **90 – 75 %** | **75 – 50 %** | **50 – 25 %** | **25 – 10 %** | **10- 0 %** |
| --- | --- | --- | --- | --- | --- | --- |
| … Nutrition | □ | □ | □ | □ | □ | □ |
| … Drugs | □ | □ | □ | □ | □ | □ |
| … Dietary supplements | □ | □ | □ | □ | □ | □ |

**What difficulties do you see in regularly addressing the consumption of food supplements during health screenings?**

| □ | Time pressure | □ | No reliable information (for patients) |
| --- | --- | --- | --- |
| □ | own insecurity | □ | unimportant topic (for myself) |
| □ | little experience | □ | more important problems when meeting patients |
| □ | Own uncertainty regarding the benefits/risks of food supplements |  |  |
| □ | Other: |  |  |

**Do patients generally report on their own initiative about food supplement consumption?**

| **always** | **often** | **rarely** | **never** |
| --- | --- | --- | --- |
| □ | □ | □ | □ |

**Which patients are more likely to report NEM consumption?** *(Multiple selection possible)*

| **Age:** | □ | Teenagers | □ | Adults |
| --- | --- | --- | --- | --- |
| **Sex:** | □ | Women | □ | Men |
| **Physical activity:** | □ | Physically active people | □ | Physically inactive people |
| **Education level:** | □ | People with a high level of education | □ | People with a low level of education |

**How often do patients have questions about food supplements?**

| **always** | **often** | **rarely** | **never** |
| --- | --- | --- | --- |
| □ | □ | □ | □ |

- **What do the patients' questions relate to?**

| □ | Intake / Dosage | □ | Effect |
| --- | --- | --- | --- |
| □ | Interactions with medications | □ | Side effects |
| □ | Safety | □ | Other _______________________ |

**Why do you think patients take food supplements?** *(Multiple selection possible)*

| □ | Maintaining health | □ | Supporting the immune system |
| --- | --- | --- | --- |
| □ | Improvement of well-being | □ | Therapy of diseases |
| □ | Nutritional support | □ | Improving your own beauty |
| □ | Improving your own quality of life | □ | Favouring alternative therapy concepts |
|  |  | □ | Other: ______________________ |

**What reasons do you think patients might have to conceal the consumption of food supplements from doctors?** *(Multiple selection possible)*

| □ | No direct request from the doctor/staff | □ | Doctor could react negatively |
| --- | --- | --- | --- |
| □ | Other problem during consultation | □ | Patient has enough information |
| □ | Time frame limited | □ | Topic unimportant |
| □ | Doctor not open to the topic | □ | Patient unprepared |
| □ | Patient thinks doctor already knows about it | □ | Irregular use |
| □ | prolonged use | □ | Doctor does not seem competent to patient with regard to food supplements |
|  |  | □ | Other: ________________________ |

**Do you routinely offer level checks for vitamins and/or minerals?**

| □ **yes** | □ **no** |
| --- | --- |

**Do you inform patients about potential side effects/interactions of food supplements?**

| □ **yes** | □ **no** |
| --- | --- |

**If yes: How long do you need on average to provide information?**

| **< 1 Minute** | **1-2 Minutes** | **3-5 Minutes** | **> 5 Minutes** |
| --- | --- | --- | --- |
| □ | □ | □ | □ |

**How often do you check the intake of prescribed food supplements?**

| **monthly** | **quarterly** | **half-yearly** | **annually** | **at the next check-up** | **never** |
| --- | --- | --- | --- | --- | --- |
| □ | □ | □ | □ | □ | □ |

- **How do you monitor the intake of food supplements?**

| □ | Anamnesis | □ | Blood sample (if possible) |
| --- | --- | --- | --- |
| *□* | Checking compliance | □ | Other ____________________ |

**How could communication about food supplements be improved in general?**

Finally, we would like to ask you to enter your background, education and experience. This data will be pseudonymised as indicated and of course treated confidentially.

**Background**

| Sex |  female  male |  divers |
| --- | --- | --- |
| Year of birth | ______ |  |
| Marital status |  single   married |  divorced   widowed |
| Nationality | ____________________ |  |
| Federal state | ____________________ |  |

**TRAINING and PRACTICAL WORK**

| Graduation year (Format YYYY) | _________________ |  |
| --- | --- | --- |
| Alma Mater | __________________ |  |
| Speciality |  Yes  designation  _______________________  _______________________  _______________________ |  No:  assistant physician (year): _____ |
| Additional designation(s) |  Naturopathy   Sports medicine   Homeopathy   Occupational medicine   Palliative care |  Acupuncture   Chirotherapy   Emergency medicine   Psychotherapy   Other: _____________ |
| Practical experience  (in years) | _____________________ |  |
| Form of practice |  solo practice   Group practice   Medical center   Hospital |  |
| Practice location |  Large city (>100.000)   Small town (>20.000)   Rural area (<20.000) |  |
| Patients |  practice financed by stationary health care |  private practice   Inpatients |

And last but not least

Would you like to be informed about the results of the study? We will be happy to send you the results by e-mail after publication of the data.

If you do not wish this, simply leave the field blank.

Please enter your e-mail address here.

Do you have any comments on dietary supplements in general practice that have not been covered or not sufficiently covered in the questionnaire?

Please let us know!

Thank you very much for your support!

Do you have any questions? We look forward to hearing from you.

**Contact:**

**Sascha Eickmann**

Telefon: XXXX

E-Mail: XXXX
